# Supplementary material for: Efficient transduction and optogenetic stimulation of retinal bipolar cells by a synthetic adeno-associated virus capsid and promoter
Source: EMBO Mol Med. 2014 Aug 4;6(9):1175–90. doi: 10.15252/emmm.201404077 (PMC4197864; doi:10.15252/emmm.201404077)
Supplement: Supplementary file 1 — Supplementary Figures [file emmm0006-1175-SD1.pdf]

TABLE OF CONTENTS

Figure S1: Genetic map of the plasmids pREDCap- preLib(S1a) and pREDCap- Lib (S1b), used to construct the AAV library.

Figure S2: Sequence changes and predicted secondary structure changes of selected AAV2/8 variants. Stick model of the secondary structure of AAV8 capsid protein with the epitope of interest highlighted in pink and the region targeted for mutation enclosed in a rectangle (S2a) and magnified (S2b). Three-dimensional structural co-ordinates of the AAV8 capsid was retrieved from Protein Data bank (PDBID:2QA0). The default orientation with stick representation is shown. Zoom selection was from aa431 (S) to 466 (Q) AND from aa572 (V) to aa606 (M). Altered sequences shown in electropherogram for AAV8BP1 (S2c), AAV8BP2(S2e), AAV8BP3(S2g) and AAV8BP4 (S2i). Predicted secondary structure changes with the associated residues in black shown for AAV8BP1 (S2d), AAV8BP2(S2f), AAV8BP3(S2h) and AAV8BP4 (S2j).Most common amino acid rotamer arising in a protein was used for the mutagenesis.

Figure S3: Microtiter data for AAV2/8BP variants used to normalize volumes in luminescence assay. Capsid genes were cloned in an AAV packaging plasmid for vector production, and used for small-scale vector preparations encoding firefly luciferase. Physical particle titers were established by TaqMan qPCR.

Figure S4: Images from mouse retinas injected with the selected AAV2/8 variants with a constitutive promoter construct. 20X confocal images from retinal sections of mice subretinally and intravitreally injected with AAV2/8BP1(EF1α-EGFP), (S4a,S4b), AAV2/8BP3(EF1α-EGFP), (S4c,S4d) and AAV2/8BP4(EF1α-EGFP), (S4e,S4f). The retinas are stained for EGFP (green), cell nuclei (grey), and for the inner plexiform layer strata using choline acetyltransferase (ChAT) (magenta). Variant 1 shows fluorescence evident in all cell types (fig. S4a,b). Variant 3 virus showed dense staining along the OPL with distinctive labeling of the horizontal cell bodies and their dense network of spreading dendrites as well as significant expression in the GCL (fig. S4c,d). The lack of staining in the ONL suggests that this variant selects against transduction of the photoreceptor cells. Variant 4 staining was sparse with only a few horizontal amacrine cells and ganglion cells labeled (fig. S4e,f). POS=Photoreceptor outer segments, ONL=Outer nuclear layer, OPL= outer plexiform layer, IPL= inner plexiform layer, GCL=Ganglion cell layer.

Figure S5: In vitro analysis of the 4xGRM6 promoter
Mouse pups were electroporated with the 4xGRM6-ChR2d-EGFP plasmid at P0. The mice were sacrificed at P21 and confocal images taken of immunostained wholemount retina. The orthogonal view is shown. The retinas are stained for EGFP (green), cell nuclei (grey), and for the inner plexiform layer strata using choline acetyltransferase (ChAT) (magenta). OPL= outer plexiform layer, IPL= inner plexiform layer, GCL=Ganglion cell layer.

Figure S6: AAV2/8BP2(4XMG6-ChR2d-EGFP)
Specific staining of ON-bipolar cells is shown in wild-type mouse retina subretinally injected with a 2μl volume of a 7×12gc/ml stock of AAV2/8BP2(4XMG6- EGFP). The EGFP protein expression is shown in green, cell nuclei are shown in blue (Hoechst), and ChAT stain is shown in magenta.

Figure S7: Rhodopsin expression in cells transduced with AAV viruses.
RT-qPCR for rhodopsin expression shows the absence of expression of rhodopsin in AAV2/8BP2(4xGRM6-ChR2d-EGFP) retinas.

Figure S8: AAV2/8BP2 transduces human ON bipolar cells (as well as photoreceptors and ganglion cells).Confocal images from a cryosection from a postmortem human retina that had been infected with AAV2/8BP2-CMV-GFP showing (left) GFP-positive cells and (right) cells labeled by immunofluorescence with Goα (red). Arrowheads indicate ON bipolar cells, i.e. GFP-positive cells (green) that were also Goα-positive (red). Note that there are also GFP-positive photoreceptors and ganglion cells. DAPI labels the nuclei (blue).
Scale bar 20 μm.

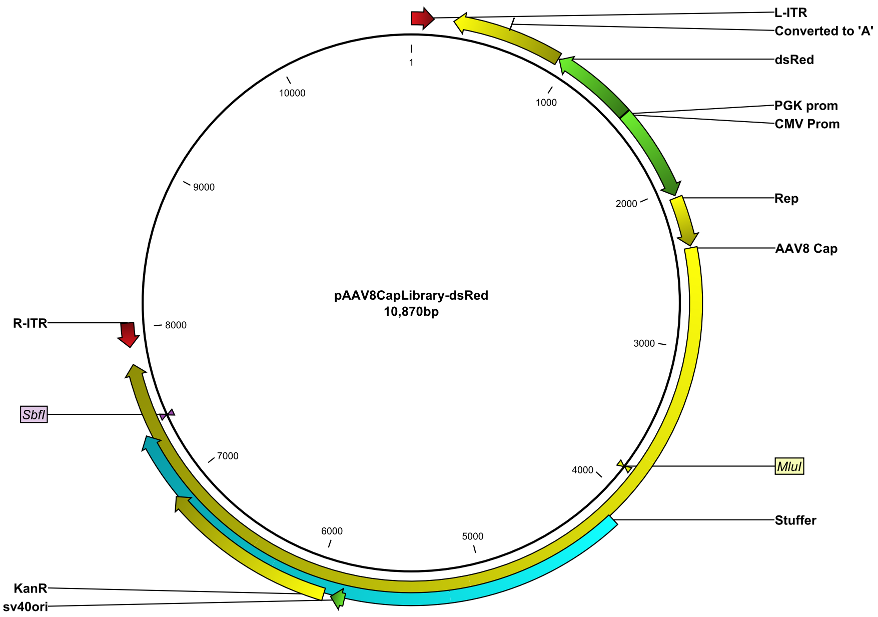

Figure S1a

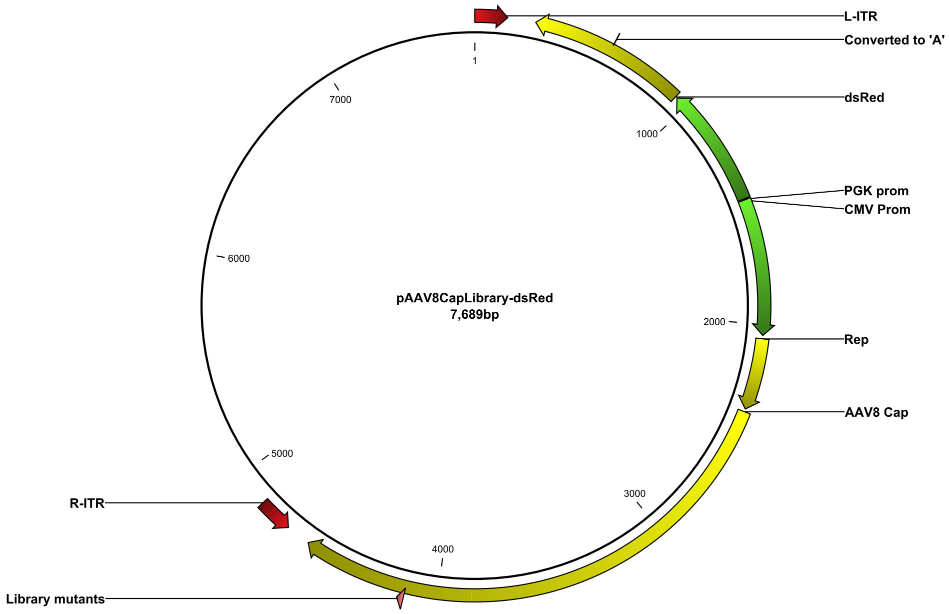

Figure S1b

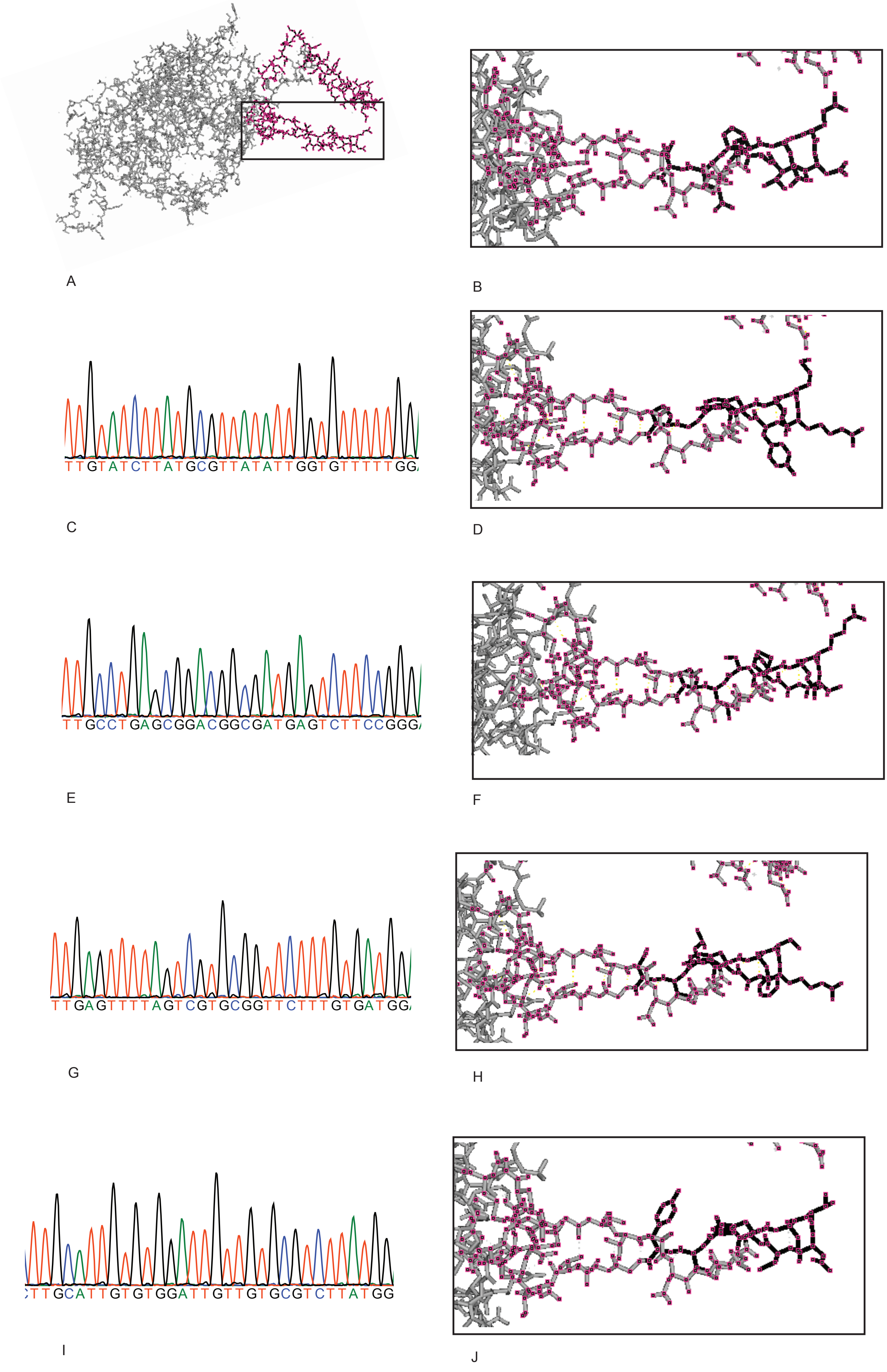
A
B
TTGTATCTTATGCGTTATATTGGTGTTTTTGG
C
D
TTGCCTGAGCGGACGGCGATGAGTCTTCCGGG
E
F
TTGAGTTTTAGTCGTGCGGTTCTTTGTGATGG
G
H
TTGCATTGTGTGGATTGTTGTGCGTCTTATGG
I
J

Figure S2

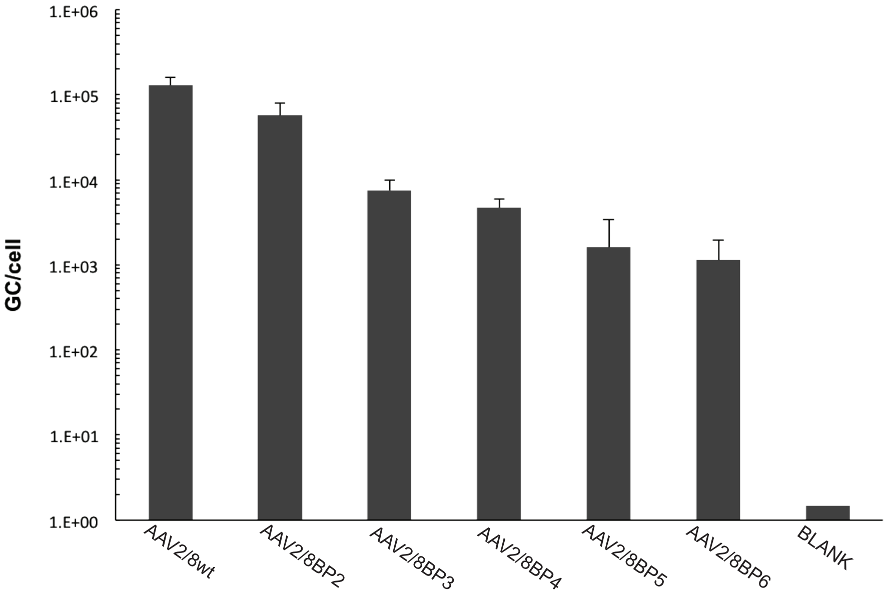
1.E+06
1.E+05
1.E+04
1.E+03
1.E+02
1.E+01
1.E+00
GC/cell
AAV2/8wt
AAV2/8BP2
AAV2/8BP3
AAV2/8BP4
AAV2/8BP5
AAV2/8BP6
BLANK

Figure S3

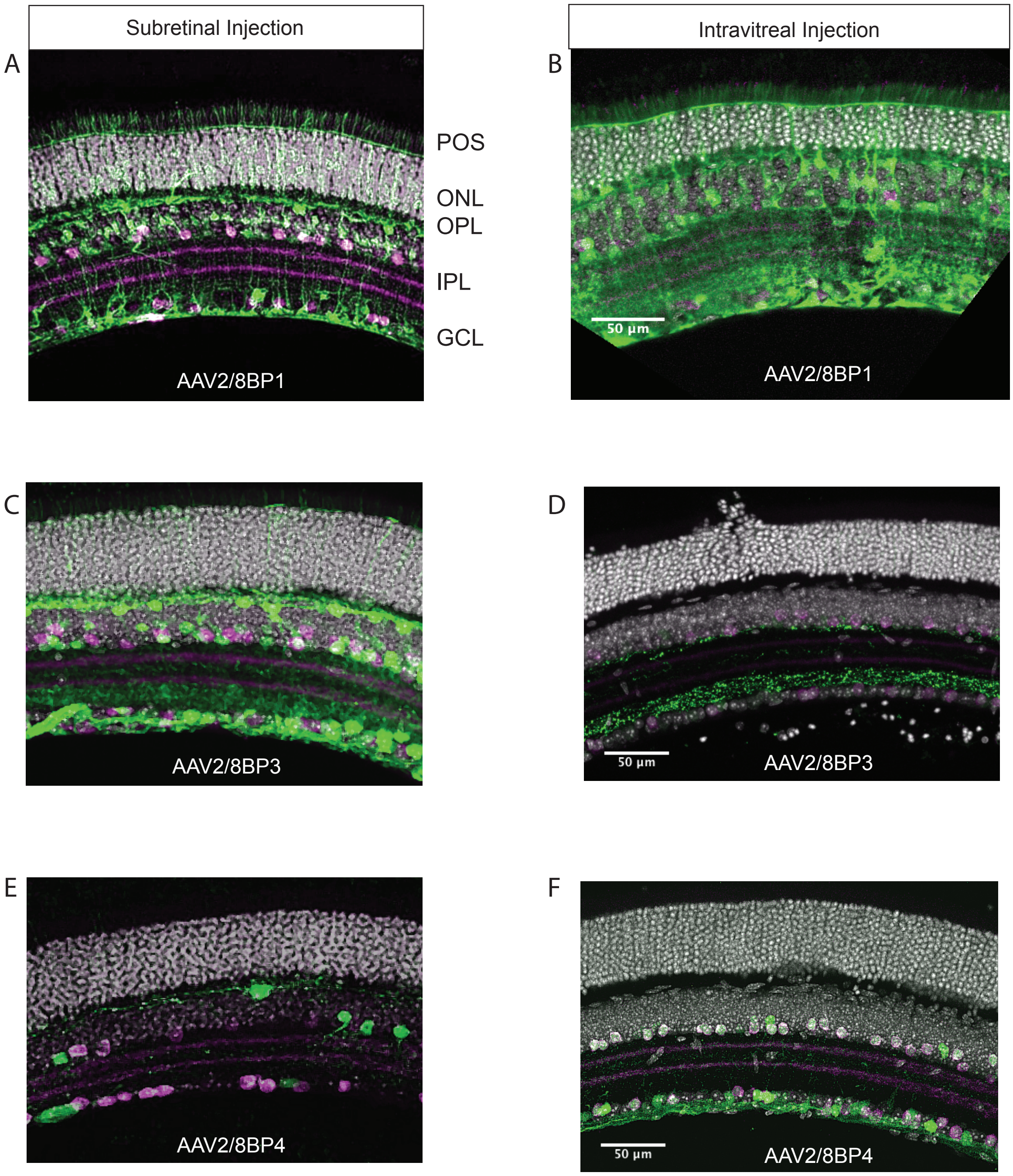
Subretinal Injection
Intravitreal Injection
A
B
POS
ONL
OPL
IPL
GCL
50 µm
AAV2/8BP1
AAV2/8BP1
C
D
AAV2/8BP3
50 µm
AAV2/8BP3
E
F
AAV2/8BP4
50 µm
AAV2/8BP4

Figure S4

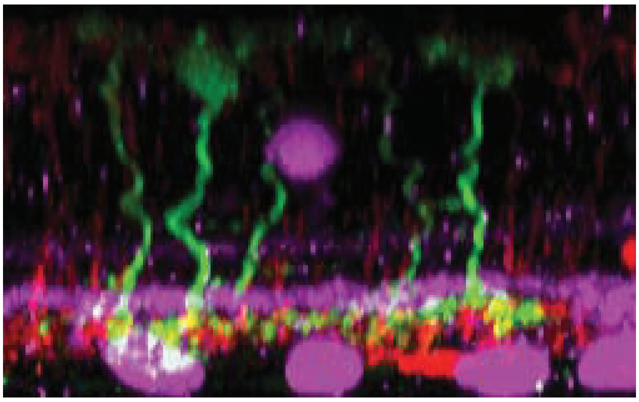

Figure S5

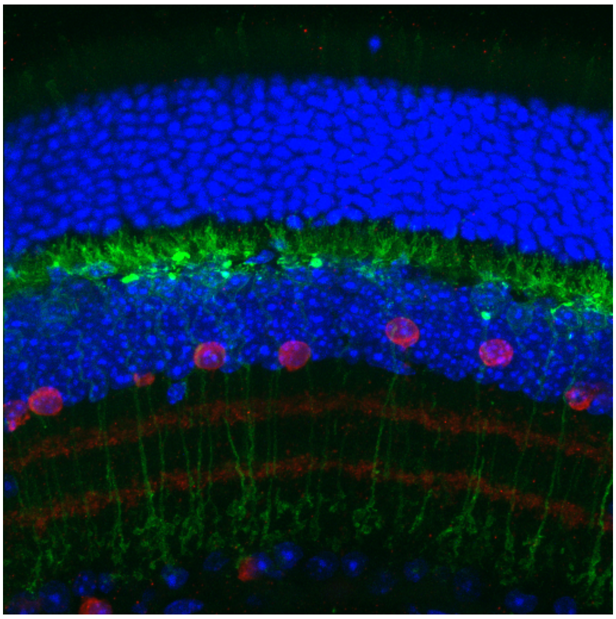

Figure S6

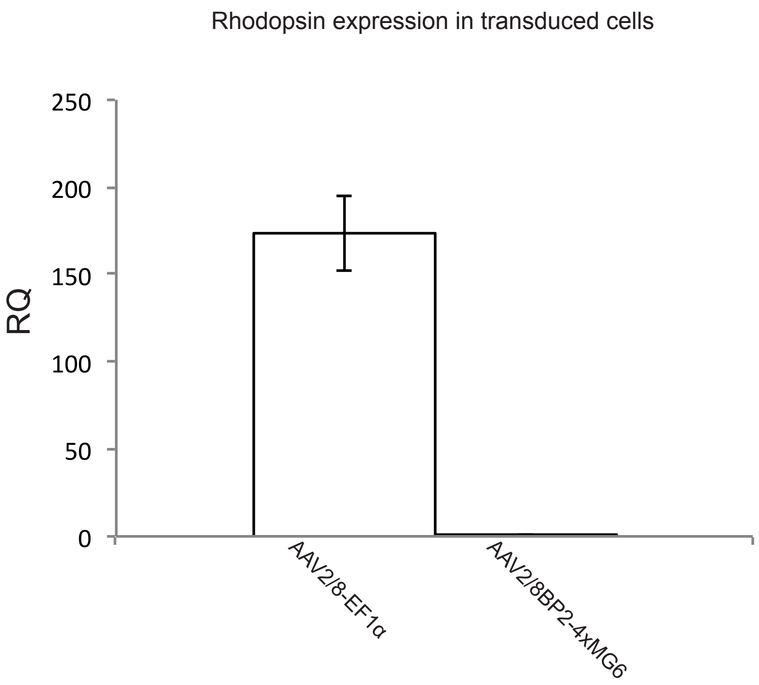
Rhodopsin expression in transduced cells
RQ
250
200
150
100
50
0
AAV2/8-EF1α
AAV2/8BP2-4xMG6

Figure S7

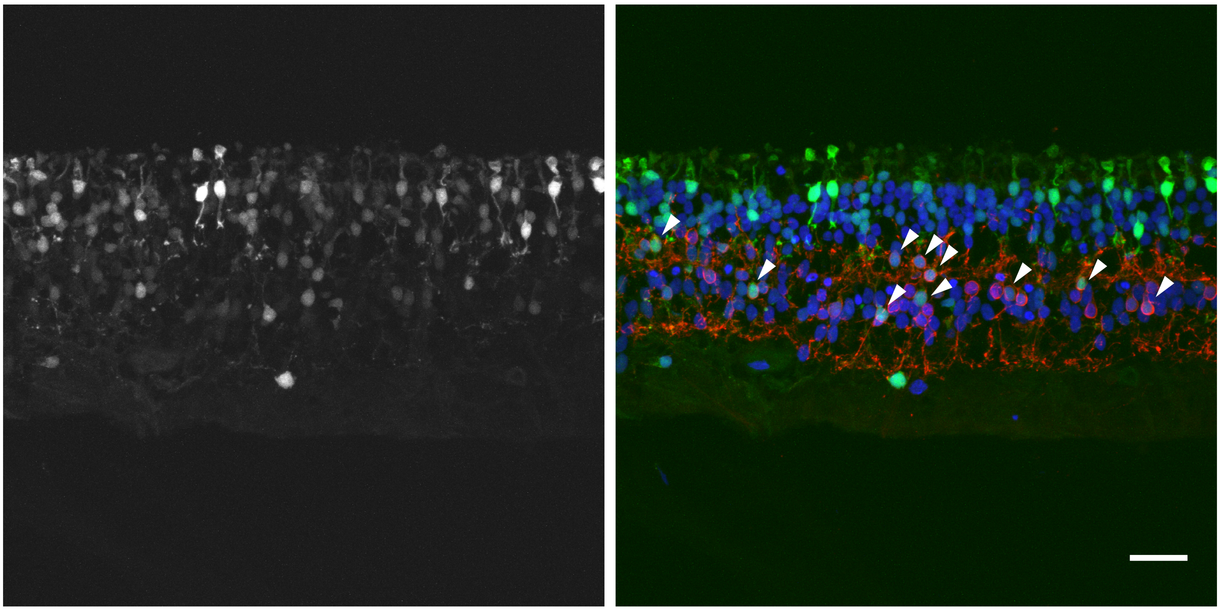

Figure S8
