# Supplementary material for: Efficient transduction and optogenetic stimulation of retinal bipolar cells by a synthetic adeno-associated virus capsid and promoter
Source: EMBO Mol Med. 2014 Aug 4;6(9):1175–90. doi: 10.15252/emmm.201404077 (PMC4197864; doi:10.15252/emmm.201404077)
Supplement: Supplementary file 2 — Supplementary Tables [file emmm0006-1175-SD2.pdf]

SUPPLEMENTARY TABLES

Primer name	Forward	Reverse	Probe
GRM6 Enhancer	CGCCGTCTAGAGCACCCAATA TTGATCTCCAGATGGCTAAACT	CTGCTAAGTCGACCAACCAGTCTT GTTTGAGCC	
AAV titer	GGCTGTTGGGCACTGACAA	CCAAGGAAAGGACGATGATTTC	TCCGTGGTGTTGTCG
Kcng4	TTCCCCCTACCTCTCTGTCA	CTCTGGAAGACATGGGCATT	
Lhx4	ACAGGCCTGGACATGAGAGT	ATCCTATTGGTGTGGCCAAG	
Prkcα	GCCGTACGGGAAGTCTGTAG	TTGGACAAGGATTTGGGGTA	
18s	GTAACCCGTTGAACCCCATT	CCATCCAATCGGTAGTAGCG	

Table S1

Gene name	TaqMan assay ID (Life Technologies)
Grm6	Mm00841148
TrpM1L	Mm01317883
Rho	Mm01184405
Opnswl	Mm00432058
Opnmwl	Mm00433560
Actb	Mm00607939

Table S2
